# Well-being inequities in eleven rural Georgia communities: A latent profile analysis

**DOI:** 10.1371/journal.pone.0320222

**Published:** 2025-04-29

**Authors:** April K. Hermstad, Regine Haardörfer, Kimberly Jacob Arriola, Peter Stoepker, Emily L. Leung, Michelle C. Kegler

**Affiliations:** 1 Rollins School of Public Health, Emory University, Atlanta, Georgia, United States of America; 2 Laney Graduate School, Emory University, Atlanta, Georgia, United States of America; 3 College of Health and Human Sciences, Kansas State University, Manhattan, Kansas, United States of America; Cranfield University, UNITED KINGDOM OF GREAT BRITAIN AND NORTHERN IRELAND

## Abstract

Personal well-being is a broad term that encompasses life domains that are not strictly health related, including social, emotional, financial, and environmental factors. Well-being is highly correlated with a range of sociodemographics and health outcomes. Little is known about well-being in rural communities, although the literature suggests that rural communities may be structurally and demographically disadvantaged regarding well-being. This cross-sectional study examined sociodemographic and health-related differences in key well-being domains, using the Personal Well-being Index (PWI). Latent Profile Analysis was used to identify demographic traits and health-related factors associated with differing levels of well-being. As part of a cross-site evaluation of a rural health initiative, we conducted a random, population-based mail survey in 11 rural counties in Georgia (USA). Between December 2018 and June 2019, surveys were mailed to 10,621 households, with 2,788 individuals completing the survey (26.2% response rate). We observed consistent differences in PWI scores and for each domain comprising the PWI across all sociodemographic categories, with characteristics that have historically conferred advantages in the USA (e.g., male gender, White race) generally being associated with greater well-being. The Latent Profile Analysis revealed five distinct well-being profiles reflecting different levels of well-being, with most respondents falling into the Very High or High well-being profile groups. Despite structural and demographic disadvantages, well-being and its sociodemographic and health-related correlates in this rural sample were comparable to that of urban settings. Coalitions working in partnership with researchers and evaluators may be an effective mechanism for identifying the factors that influence well-being and health. Interventions on social determinants of health may help determine what approaches are successful and best promote well-being, in addition to health, while also reducing socioeconomic inequities.

## Introduction

In 1946, the World Health Organization (WHO) defined health as “a state of complete physical, mental and social well-being,” centering well-being as a criterion for health [1]. Indeed, positive well-being may be protective for physical health, especially with aging [2]. While the WHO definition of health remains frequently cited today, contemporary scholars argue that health may be better viewed as a component of well-being. Health metrics typically emphasize disease states, morbidity, and mortality. The lack of well-being measures used in assessing health further supports the argument that health and well-being are separate, though potentially interrelated, constructs [3,4]. Healthy People 2030’s elevation of well-being to a primary outcome may signal a shift toward broader metrics for health and quality of life [4].

Well-being is a broad term encompassing life domains that are not strictly health related, including social, emotional, financial, and environmental factors [5]. The culmination of these factors is a global evaluation of one’s life [6]. A single-item measure of overall life satisfaction is more commonly used to assess well-being than measures of separate domains [5,7–12]. Flourishing, often used interchangeably with subjective well-being [11], is a composite measure of emotional, social, and psychological well-being [13,14].

Descriptive studies of U.S. populations have found greater levels of flourishing among men compared to women [14] and greater life dissatisfaction among women [8]. Studies in other western nations have found the opposite [7,10]. Reduced life satisfaction is associated with identifying as Black, American Indian/Alaska Native, and Hispanic, with only Asian adults reporting greater levels of life satisfaction than White adults [8–9]. Emotional well-being has also been found to be lower among Black or Hispanic adults than White adults. Keyes & Simoes [14] found no significant difference in flourishing between Black and White adults. Greater life satisfaction is associated with being married, greater educational attainment, employment, and higher incomes [8–11,13,14]. Similarly, flourishing is associated with being male, married, and having more years of education [13]. Well-being appears to have a U-shaped relationship with age, where life satisfaction tends to reach the nadir in middle age [2,8,9]. Keyes [13,14] found that flourishing was highest among older adults. Cummins [7] found that certain dimensions of well-being are lowest at different stages of life, with younger adults dissatisfied with their community connections and personal relationships, middle-age groups dissatisfied with their future security, and older adults dissatisfied with their health.

Measures of well-being are negatively associated with health behaviors including smoking, alcohol use, and physical inactivity [8,14–16]. Similarly, well-being has been inversely associated with health outcomes including several chronic conditions (e.g., asthma, diabetes, heart disease, hypertension, obesity), poor or fair health status, poor physical and mental health, and activity limitations [8,9,13,14,17].

There is scant evidence regarding the relationship between rurality and well-being. In the U.S., Baernholdt [17] found no differences in emotional well-being between urban and rural dwellers. In an Australian sample, Cummins [7] found that people living in lower access, more remote areas reported greater levels of life satisfaction than those in higher access, less remote areas. In the U.S., rural communities possess many strengths, including social cohesion and connectedness, innovation, creative problem solving, and resilience [18]. Despite these important assets, numerous social and economic drivers have contributed to rural communities having population-level demographic traits associated with poorer self-reported well-being [19–22]. Rural places have been slower to recover from the Great Recession than urban areas, due in part to less workforce investment in rural areas, resulting in fewer employment opportunities [19,20,23]. Furthermore, rural areas tend to have low and declining tax bases, which limits public spending on social services and social determinants of health (SDOH) [20,23]. Rural areas face disparities in numerous SDOH including access to health care and longer distances to travel to receive care [19,22], access to healthy foods [20,22,23], physical activity opportunities [22], housing quality and availability, education [20], and transportation access [23]. As in urban communities, systemic racism has produced differential access to resources and opportunities across racial lines within rural communities [24–25].

Rural demographics differ from metropolitan and urban areas in several important ways. Rural communities are often older with higher proportions of elderly residents, have lower incomes and higher rates of poverty, lower levels of educational attainment, and higher rates of disability, uninsured, and food insecurity [19–23]. As rural areas grow more racially diverse, these differences are compounded by the fact that racial and ethnic minority groups tend to experience less favorable socioeconomic conditions than their White counterparts [19,23]. In addition, rural communities tend to have higher rates of modifiable risk factors for disease (e.g., tobacco use, physical inactivity) and worse health (e.g., self-rated health status, chronic diseases) and mortality outcomes than urban areas [21,23].

Our earlier work has documented sociodemographic and health inequities within rural communities [26–29]. Yet, the relationship between well-being and sociodemographic and health factors in rural communities is not established. The current descriptive study uses data from a health equity initiative in 11 rural Georgia counties to examine sociodemographic and health-related differences in key well-being domains. Additionally, we used Latent Profile Analysis to identify demographic and health-related factors associated with differing levels of well-being. Findings may inform programs and policies to address the specific needs of rural populations in the U.S.

## Materials and methods

### The Two Georgias Initiative

*The Two Georgias Initiative* was a 5-year, place-based grantmaking initiative (2017–2022) designed to address health equity among rural Georgia communities. Healthcare Georgia Foundation developed the initiative and funded 11 rural health coalitions. Initiative goals were to achieve greater health equity among rural populations, improve health and health care for rural Georgians, build healthier rural communities, improve social conditions impacting health of rural populations, and build community, organizational, and individual leadership capacity in rural Georgia.

### Study design, respondents, and recruitment

This cross-sectional study uses data from a baseline population-based mail survey conducted as part of the cross-site evaluation [30]. The survey explored concepts related to common coalition priorities, with different modules administered in each community depending on local priorities (i.e., food access, dietary behavior, physical activity environments and behaviors, health care access and use, social capital) and tailored for each coalition community. All surveys included modules for health-related quality of life, well-being, and demographics. Eligible respondents were adults living in a household receiving the mail survey. The Emory University Institutional Review Board (IRB) determined that this study was a non-research program evaluation not requiring IRB approval.

Surveys were mailed between December 7, 2018 and June 18, 2019–10,621 randomly selected households across the 11 coalition counties. Each mailing included a letter describing the study and incentive, a survey, and a stamped return envelope. Although informed consent was not required for this study, we provided participants with information similar to what would be included in an informed consent form (e.g., participation was completely voluntary, confidential recordkeeping) in the mailed materials and return of the survey was taken to indicate their agreement to participate. Within a month of the initial survey mailing, a postcard reminder about the survey followed by a second survey mailing was sent to households that had not yet responded. A total of 2,788 completed surveys (26.2% response rate) were received between December 14, 2018 and August 7, 2019. Because respondents were informed they could skip any question they did not want to answer, we defined a complete survey as one with ≥50% of questions answered, regardless of which questions were answered or left blank. The time commitment for this study was approximately 15 minutes. Respondents who returned a completed survey received a $15 gift card.

### Measures

#### Well-being.

Well-being was assessed using the Personal Well-being Index (PWI) [31]. This instrument asks respondents to rate their satisfaction with eight life domains: (a) standard of living, (b) personal health, (c) life achievements, (d) personal relationships, (e) personal safety, (f) feeling part of community, (g) future security, and (h) spirituality or religion. The rating scale ranged from zero (no satisfaction) to 10 (complete satisfaction). For analysis, the eight life domains were averaged to create the PWI scale, consistent with protocol [31].

#### Food security.

Food security was assessed using a validated 2-item measure [32] that asked respondents to indicate how often (never, sometimes, often) in the past 12 months they “worried whether my food would run out before I got money to buy more” and “the food that I bought just didn’t last and I didn’t have money to get more.” Respondents who answered sometimes or often to either item were considered food insecure.

#### Body mass index (BMI).

Respondents were asked “About how tall are you without shoes?” and space was provided to answer in feet and inches. Respondents were also asked “About how much do you weigh without shoes?” and provided their weight in pounds. We used the Centers for Disease Control and Prevention’s formula for calculating BMI based on self-reported height and weight [33]. Continuous BMI values were classified into three groups: healthy weight (18.5–24.9), overweight (25.0–29.9), and obese (≥30.0). Values outside of 18.5–60 were excluded.

#### Health status.

A standard item [34] was used to assess general health status using the response options poor, fair, good, very good, and excellent, which were dichotomized into poor/fair and good/very good/excellent.

#### Demographics.

These measures were adapted from the Behavioral Risk Factor Surveillance System [34] or the U.S. Census Bureau’s American Communities Survey [35]. Questions assessing individual and household characteristics included age, gender, race and ethnicity, marital status, educational attainment, employment status, annual household income, neighborhood type/rurality, and household size. Response options for several variables were recoded for analysis. Table 1 presents complete demographic questions and response options.

**Table 1:** Survey Demographic Questions and Response Options.

Variable	Question	Response options
**Age**	How old are you?	[open-ended]
**Gender**	What is your gender?	Female Male
**Race**	What is your race or ethnicity?	White, not of Hispanic origin African American or Black, not of Hispanic origin Hispanic More than one race Other, please specify
**Marital status**	What is your marital status?	Married Not married, living with a partner In a relationship, not living together Separated Divorced Widowed Single
**Educational attainment**	What is the highest grade or year of school you completed?	8^th^ grade or less Some high school High school or GED certificate Some college or technical school College graduate Post-graduate or professional degree
**Employment status**	What is your employment status?	Working, full-time Working, part-time Retired Not employed/Homemaker/Student/On disability Other, please specify
**Annual household income**	What is your total yearly household income from all sources?	$10,000 or less $10,001-$15,000 $15,001-$20,000 $20,001-$25,000 $25,001-$35,000 $35,001-$50,000 $50,001-$75,000 More than $75,000 Prefer not to answer
**Neighborhood type/rurality**	Which of the following best describes the neighborhood where you live? By neighborhood, we mean the area within about a 20-minute walk from your home.	In town In the country or rural area with neighbors close by In the country or rural area with very few neighbors close by
**Household size**	Including yourself, how many people live in your household? (Count anyone who sleeps there 3 times or more per week.)	[open-ended]

### Data analysis

We used SPSS 28 to run frequencies and descriptive statistics for all demographic variables, the PWI scale, and eight items that comprise the index. Analysis of variance and t-tests were used to explore demographic group differences for the PWI. The Bonferroni correction was used to account for multiple comparisons. We conducted a Latent Profile Analysis on the well-being items using Mplus 8.7. LPA is a person-centered approach that allows groups to emerge from indicators that are hypothesized to “hang together” or show distinct patterns (i.e., profiles) rather than from group membership based on sociodemographic or other grouping variables. We ran a series of models to identify the best number of profiles from 1 to 8 profiles. We used entropy, Akaike Information Criterion, Bayesian Information Criterion, sample size adjusted Bayesian Information Criterion, and smallest class sizes to identify the final set of profiles. Subsequently, we used SAS 9.4 to conduct a multinomial binary logistic regression with profile membership as the outcome and key variables for this study to understand characteristics of respondents and profile membership. We did not run a multivariable multinomial model as the missingness across sociodemographic variables would have reduced the analytic sample too much and multiple imputations did not seem viable for variables such as race and marital status. The threshold for significance was.05.

## Results

### Respondent demographics

Most respondents were 35–64 years of age (50.9%) or ≥65 years (41.3%), female (66.3%), and married (54.9%) (Table 2). A majority were White (78.8%) and 21.2% were Black or African American. Educational attainment varied, with 10.7% reporting some high school or less, 28.0% reporting a high school diploma or GED certificate, 31.3% with some college or technical school, and 29.8% reporting a college degree or higher. A large proportion of respondents were retired (39.2%) and 36.5% worked full-time. Annual household income was somewhat evenly distributed, with 28.4% reporting ≤$20,000 and 34.9% reporting incomes>$50,000. About half of respondents lived in a rural area with neighbors close by (50.8%), and a quarter lived in-town (24.4%). For household size, 24.4% lived alone, 44.5% lived with one other person, and 31.1% lived with two or more people. Nearly half (45.2%) reported food insecurity in the past 12 months. About four in ten respondents (40.9%) were obese and about a quarter (25.6%) reported poor/fair health.

**Table 2:** Survey Respondent Demographic Descriptives (n = 2,788).

Demographics	n (%)
**Age (years)**	
18-34	212 (7.9%)
35-64	1370 50.9%)
65	1112 (41.3%)
**Gender**	
Male	910 (33.7%)
Female	1794 (66.3%)
**Race**	
White	2048 (78.8%)
African American/Black	552 (21.2%)
**Marital status**	
Married	1485 (54.9%)
Not married	1220 (45.1%)
**Educational attainment**	
Some high school	295 (10.8%)
High school or GED	763 (28.0%)
Some college or technical school	853 (31.3%)
College graduate and above	812 (29.8%)
**Employment status**	
Working, full- time	960 (36.5%)
Working, part- time	224 (8.5%)
Retired	1031 (39.2%)
Not employed/homemaker/student/on disability	417 (15.8%)
**Annual household income**	
≤$20,000	639 (28.4%)
$20,001-$50,000	824 (36.7%)
≥$50,001	784 (34.9%)
**Rurality**	
In town	706 (25.9%)
Rural area, neighbors close by	1385 (50.8%)
Rural area, very few neighbors close by	633 (23.2%)
**Household size**	
Lives alone	657 (24.4%)
Lives with 1 other	1200 (44.5%)
Lives with ≥2 others	838 (31.1%)
**Food security**	
Secure	1297 (54.8%)
Insecure	1068 (45.2%)
**BMI**	
Healthy (18.5–24.9)	649 (25.0%)
Overweight (25.0–29.9)	883 (34.1%)
Obese (≥30)	1059 (40.9%)
**Health status**	
Poor/fair	698 (25.6%)

### PWI and item means by demographic and health characteristics

Tables 3 and 4 show means for the PWI (7.6) and individual items overall and differences by demographic and health groups. Item means ranged from 6.9 (feeling part of community) to 8.5 (spirituality or religion). There were significant differences between all sociodemographic and health variables for the PWI and nearly all individual well-being items (Tables 3 and 4). Bivariate analyses found certain groups consistently reported statistically significantly greater PWI and individual item scores, including those who were age ≥65, male, White, married, college graduates, retired, had higher incomes, and living in a rural area with neighbors close by. PWI scores were also significantly greater among those who were food secure, were not obese, and reported good or better health status.

**Table 3:** Mean and Standard Deviation for Well-being Items by Sociodemographic Characteristics (n = 2,788).

Characteristic	PWI Scale (a-h)	(a) Standard of living	(b) Your health	(c) Achieving in life	(d) Your personal relationships	(e) How safe you feel	(f) Feeling part of community	(g) Your future security	(h) Your spirituality or religion
**Overall sample**, Mean (SD)	7.6 (1.9)	7.7 (2.3)	6.9 (2.4)	7.2 (2.5)	7.8 (2.5)	8.2 (2.1)	6.9 (2.8)	7.1 (2.7)	8.5 (2.1)
**Age**, Mean (SD)	**<.001**	**<.001**	**<.001**	**<.001**	**<.001**	**.005**	**<.001**	**<.001**	**<.001**
18-34	7.4 (1.8)	7.3 (2.4)	7.2 (2.2)	7.3 (2.3)	8.1 (2.2)	8.5 (2.0)	6.2 (2.8)	6.8 (2.6)	8.1 (2.1)
35-64	7.3 (2.0)	7.4 (2.4)	6.7 (2.5)	7.0 (2.6)	7.6 (2.6)	8.1 (2.2)	6.6 (2.8)	6.7 (2.8)	8.3 (2.2)
≥65	8.0 (1.8)	8.1 (2.0)	7.2 (2.4)	7.5 (2.5)	8.0 (2.3)	8.4 (2.0)	7.4 (2.6)	7.7 (2.4)	8.9 (1.8)
**Gender**, Mean (SD)	**<.001**	**.015**	.338	**.002**	**.002**	**<.001**	**<.001**	**<.001**	.964
Male	7.8 (1.8)	7.8 (2.2)	7.0 (2.4)	7.4 (2.4)	8.0 (2.2)	8.5 (1.9)	7.2 (2.6)	7.4 (2.5)	8.5 (2.0)
Female	7.5 (2.0)	7.6 (2.3)	6.9 (2.5)	7.1 (2.6)	7.7 (2.6)	8.1 (2.2)	6.8 (2.8)	7.0 (2.8)	8.5 (2.1)
**Race**, Mean (SD)	**<.001**	**<.001**	**.005**	**<.001**	**<.001**	**<.001**	**.026**	**.007**	.468
White	7.7 (1.9)	7.8 (2.2)	7.0 (2.4)	7.3 (2.5)	7.9 (2.4)	8.4 (2.0)	7.0 (2.7)	7.2 (2.7)	8.6 (2.0)
African American/Black	7.2 (2.0)	7.1 (2.4)	6.7 (2.5)	6.9 (2.6)	7.2 (2.7)	7.8 (2.3)	6.7 (2.8)	6.9 (2.7)	8.5 (2.1)
**Marital status**, Mean (SD)	**<.001**	**<.001**	**<.001**	**<.001**	**<.001**	**<.001**	**<.001**	**<.001**	**<.001**
Married	7.9 (1.7)	8.1 (2.0)	7.2 (2.3)	7.7 (2.2)	8.4 (2.0)	8.6 (1.8)	7.2 (2.6)	7.5 (2.4)	8.7 (1.9)
Not married	7.1 (2.1)	7.2 (2.5)	6.6 (2.6)	6.7 (2.7)	7.0 (2.8)	7.9 (2.3)	6.5 (3.0)	6.6 (2.9)	8.3 (2.2)
**Educational attainment**, Mean (SD)	**<.001**	**<.001**	**<.001**	**<.001**	**<.001**	**<.001**	**.002**	**<.001**	.091
Some high school or less	7.2 (2.1)	7.1 (2.5)	6.2 (2.7)	6.6 (2.9)	7.5 (2.7)	7.9 (2.3)	6.5 (3.0)	6.9 (2.9)	8.4 (2.4)
High school/GED	7.5 (2.0)	7.6 (2.3)	6.8 (2.5)	7.0 (2.7)	7.8 (2.6)	8.2 (2.2)	6.9 (2.9)	7.0 (2.8)	8.6 (2.1)
Some college/tech school	7.4 (2.0)	7.6 (2.3)	6.9 (2.4)	7.2 (2.4)	7.6 (2.5)	8.1 (2.1)	6.8 (2.8)	6.9 (2.7)	8.4 (2.1)
College and above	7.9 (1.7)	8.1 (1.9)	7.4 (2.2)	7.7 (2.2)	8.1 (2.1)	8.5 (1.9)	7.2 (2.5)	7.6 (2.4)	8.6 (1.9)
**Employment status**, Mean (SD)	**<.001**	**<.001**	**<.001**	**<.001**	**<.001**	**<.001**	**<.001**	**<.001**	**<.001**
Working, full-time	7.8 (1.6)	7.9 (1.9)	7.5 (1.9)	7.6 (2.0)	8.0 (2.2)	8.5 (1.8)	7.0 (2.5)	7.1 (2.4)	8.4 (2.0)
Working, part-time	7.7 (1.8)	7.6 (2.3)	7.2 (2.3)	7.5 (2.3)	8.0 (2.2)	8.3 (2.0)	7.0 (2.7)	7.2 (2.7)	8.5 (2.1)
Retired	7.9 (1.8)	8.0 (2.1)	7.1 (2.4)	7.4 (2.5)	7.9 (2.3)	8.3 (2.1)	7.3 (2.7)	7.6 (2.5)	8.9 (1.8)
Not employed	6.5 (2.3)	6.6 (2.8)	5.6 (2.9)	5.9 (3.0)	6.9 (3.0)	7.6 (2.6)	5.7 (3.1)	6.0 (3.2)	8.0 (2.6)
**Income**, Mean (SD)	**<.001**	**<.001**	**<.001**	**<.001**	**<.001**	**<.001**	**<.001**	**<.001**	**<.001**
≤$20,000	6.5 (2.3)	6.4 (2.7)	5.7 (2.8)	6.0 (3.0)	6.8 (2.9)	7.3 (2.6)	5.9 (3.1)	6.0 (3.1)	8.1 (2.6)
$20,001-$50,000	7.5 (1.8)	7.6 (2.1)	6.9 (2.4)	7.1 (2.4)	7.7 (2.4)	8.3 (2.0)	6.8 (2.8)	6.9 (2.7)	8.5 (2.0)
≥$50,001	8.2 (1.4)	8.5 (1.5)	7.6 (2.0)	8.0 (1.8)	8.4 (1.8)	8.8 (1.5)	7.4 (2.3)	7.8 (2.1)	8.7 (1.7)
**Rurality**, Mean (SD)	**.004**	**.009**	**.014**	**.006**	.050	**.022**	**.017**	.153	.291
In town	7.4 (2.0)	7.5 (2.4)	6.7 (2.5)	7.0 (2.7)	7.6 (2.5)	8.1 (2.2)	6.7 (2.9)	7.0 (2.7)	8.5 (2.2)
Rural area, neighbors close by	7.7 (1.8)	7.8 (2.2)	7.0 (2.4)	7.3 (2.4)	7.9 (2.3)	8.4 (2.0)	7.0 (2.7)	7.2 (2.6)	8.6 (2.0)
Rural area, few neighbors close by	7.5 (2.0)	7.7 (2.2)	7.0 (2.4)	7.3 (2.5)	7.7 (2.6)	8.1 (2.2)	6.7 (2.7)	7.0 (2.7)	8.5 (2.1)
**Household size**, Mean (SD)	**<.001**	**<.001**	.298	**<.001**	**<.001**	**<.001**	**.028**	**.005**	.067
Lives alone	7.4 (2.1)	7.4 (2.4)	6.8 (2.5)	6.9 (2.7)	7.2 (2.7)	7.9 (2.3)	6.9 (2.8)	7.0 (2.8)	8.6 (2.0)
Lives with 1 other	7.7 (1.9)	8.0 (2.1)	7.0 (2.5)	7.4 (2.5)	8.0 (2.3)	8.3 (2.0)	7.0 (2.7)	7.3 (2.6)	8.6 (2.0)
Lives with ≥2 others	7.5 (1.9)	7.5 (2.3)	7.0 (2.4)	7.2 (2.5)	7.8 (2.4)	8.4 (2.0)	6.7 (2.8)	6.9 (2.7)	8.4 (2.2)

**Table 4:** Mean and Standard Deviation for Well-being Items by Health Characteristics (n = 2,788).

Characteristic	PWI Scale (a-h)	(a) Standard of living	(b) Your health	(c) Achieving in life	(d) Your personal relationships	(e) How safe you feel	(f) Feeling part of community	(g) Your future security	(h) Your spirituality or religion
**Overall sample**	7.6 (1.9)	7.7 (2.3)	6.9 (2.4)	7.2 (2.5)	7.8 (2.5)	8.2 (2.1)	6.9 (2.8)	7.1 (2.7)	8.5 (2.1)
**Food insecurity**	**<.001**	**<.001**	**<.001**	**<.001**	**<.001**	**<.001**	**<.001**	**<.001**	**<.001**
Secure	8.3 (1.4)	8.5 (1.5)	7.6 (2.0)	8.1(1.9)	8.5 (1.9)	8.8 (1.6)	7.6 (2.3)	8.0 (2.0)	8.9 (1.6)
Insecure	6.7 (2.1)	6.6 (2.5)	6.0 (2.6)	6.1 (2.8)	6.9 (2.8)	7.6 (2.4)	6.0 (3.0)	5.9 (3.0)	8.1 (2.5)
**Body mass index**	**<.001**	**<.001**	**<.001**	**<.001**	**<.001**	**.003**	**<.001**	**<.001**	**.009**
Healthy (18.5–24.9)	7.7 (1.9)	7.8 (2.3)	7.3 (2.3)	7.4 (2.5)	7.9 (2.3)	8.3 (2.1)	7.0 (2.8)	7.3 (2.6)	8.7 (2.0)
Overweight (25.0–29.9)	7.8 (1.8)	8.0 (2.0)	7.3 (2.3)	7.5 (2.3)	8.0 (2.3)	8.4 (1.9)	7.1 (2.6)	7.3 (2.5)	8.5 (2.1)
Obese (≥30.0)	7.6 (1.9)	7.7 (2.2)	6.9 (2.4)	7.2 (2.5)	7.8 (2.5)	8.2 (2.1)	6.9 (2.8)	7.1 (2.7)	8.5 (2.1)
**Health status**	**<.001**	**<.001**	**<.001**	**<.001**	**<.001**	**<.001**	**<.001**	**<.001**	**<.001**
Poor/fair	6.2 (2.2)	6.3 (2.6)	4.5 (2.5)	5.4 (2.9)	6.6 (3.0)	7.3 (2.6)	5.5 (3.0)	5.8 (3.1)	7.9 (2.6)
Good/very good/excellent	8.0 (1.6)	8.1 (1.9)	7.8 (1.7)	7.8 (2.0)	8.2 (2.1)	8.5 (1.8)	7.4 (2.5)	7.6 (2.4)	8.7 (1.8)

### Well-being item ranks by demographic and health characteristics

**Fig 1:**
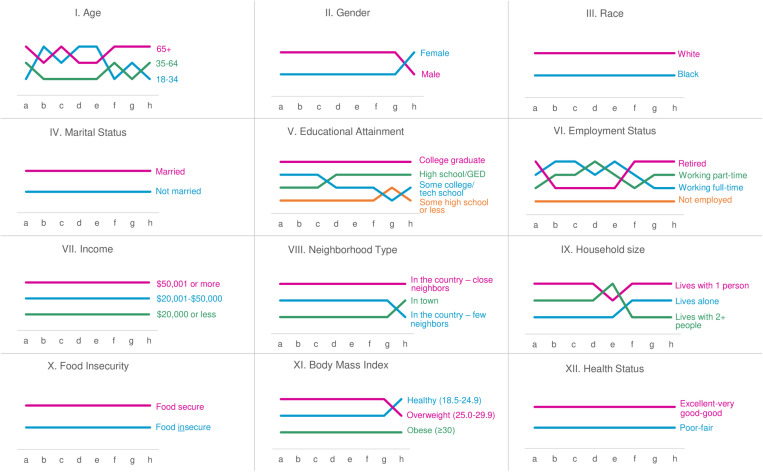
Bump charts showing relationship between eight well-being domains and 12 demographic and health characteristics. (a) Your standard of living, (b) your health, (c) what you are achieving in life, (d) your personal relationships, (e) how safe you feel, (f) feeling part of your community, (g) your future security, (h) your spirituality or religion.

Illustrating the patterns described above, Fig 1 shows 12 bump charts exploring PWI item rankings by each demographic and health-related characteristic (Roman numerals I-XII). The eight PWI items are shown along the x-axis (letters a-h), indicating relative ranks of categorical groups for the related characteristic, with higher ranks reflecting higher relative well-being scores. Crossing of the lines represents a change in rank.

About half of the charts show groups in a consistent rank with one group reporting higher mean scores for all eight well-being items than the other group(s), including I-race (White), IV-marital status (married), VII-income (>$50,000), X-food security (food secure), and XII-health status (excellent/very good/good). Other variables showed slight variation in ranks. For gender (II), neighborhood type (VIII), and body mass index (XI), one group consistently ranked higher than the other, until ranks changed for item h, spirituality or religion.

A few characteristics showed more complex relationships with well-being. For chart I-age, rankings for each group were highly variable, although the middle age group (ages 35–64) never held top rank, and those age ≥65 never held bottom rank. For chart V-educational attainment, college graduates consistently reported higher well-being scores than the two middle educational attainment groups, which largely alternated between second and third ranks. Those with the lowest educational attainment reported the lowest levels of well-being for all items except for item g (future security). For chart VI-employment status, those not employed consistently held the lowest rank, while those who were retired, working full-time, and working part-time frequently changed ranks from one item to the next. Last, chart IX-household size shows that those living with one other person held top rank for all well-being items except for item e (how safe you feel), and those living alone held bottom rank for all but 3 well-being items. Those living with 2 or more people largely occupied middle and bottom ranks, but ranked highest for well-being item e.

### Latent profile analysis

**Fig 2:**
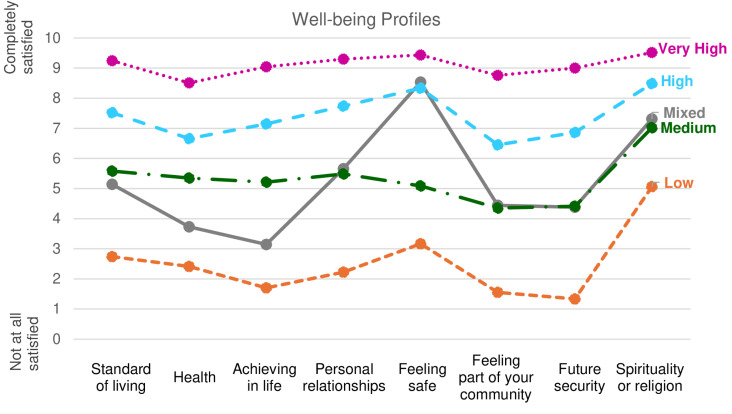
Mean values on the rating scale for items included in the well-being LPA analyses among class members. All comparisons are statistically significantly different except that Mixed versus Medium are not statistically significant for personal relationships, community, future security, and spirituality/religion.

Based on a comprehensive set of indicators, the 5-profile solution best fit the data (S1 Table). Fig 2 shows mean values for each PWI item by group, with four main profiles exhibiting similar patterns across all well-being items at distinct levels (Low, Medium, High, and Very High). The Very High profile was the largest, capturing 44.5% of respondents (mean PWI=9.1), followed by the High well-being profile (32.6%, mean PWI=7.4), Medium well-being profile (11.2%, mean PWI=5.4), and Low well-being profile (4.7%, mean PWI=2.6). The fifth group, which we refer to as “Mixed,” representing 7.1% of respondents (mean PWI=5.3), showed a different pattern than the other profiles. These members were similar to the Medium well-being profile in 5 well-being domains (standard of living, relationships, community, future security, religion) but scored lower in health and achieving in life, and were similar to the High well-being group on feeling safe (Table 5). There were significant differences in PWI scores for all groups except for Medium and Mixed. In the sections below, descriptives of sociodemographics by well-being profile reported are from Tables 5 and 6, and Odds Ratios (ORs) highlighting difference between well-being groups can be found in S2 Table.

**Table 5:** Survey Respondent Sociodemographic Descriptives by Well-being Profile (n=2,720).

Profile	Total		Very high		High		Medium		Low		Mixed	
Variable	N/mean	%/SD	N/mean	%/SD	N/mean	%/SD	N/mean	%/SD	N/mean	%/SD	N/mean	%/SD
Sample size and descriptives, n (%)	2720	100.0	1210	44.5	886	32.6	305	11.2	127	4.7	192	7.1
PWI Score, Mean (SD)	7.56	1.9	9.13	0.6	7.39	0.6	5.35	0.7	2.58	1.0	5.27	0.9
Variable	N	%	N	%	N	%	N	%	N	%	N	%
**Age**, n (%)												
18-34	213	7.9	82	6.9	91	10.4	17	5.7	11	8.9	12	6.3
35-64	1362	50.8	529	44.3	479	54.7	166	55.7	79	63.7	109	57.4
≥65	1106	41.3	583	48.8	305	34.9	115	38.6	34	27.4	69	36.3
**Gender**, n (%)												
Male	907	33.7	425	35.5	311	35.4	81	27.0	30	24.0	60	31.8
Female	1782	66.3	771	64.5	568	64.6	219	73.0	95	76.0	129	68.3
**Race**, n (%)												
White	2036	78.8	968	83.7	643	76.3	192	68.1	83	68.6	150	82.9
African American/Black	548	21.2	189	16.3	200	23.7	90	31.9	38	31.4	31	17.1
**Marital status**, n (%)												
Married	1482	55.1	761	63.6	501	57.0	115	38.3	35	28.0	70	36.8
Not married	1208	44.9	435	36.4	378	43.0	185	61.7	90	72.0	120	63.2
**Employment**, n (%)												
Working, full-time	955	36.6	443	37.7	351	41.4	98	33.9	17	14.7	46	25.7
Working, part-time	223	8.6	102	8.7	76	9.0	25	8.6	8	6.9	12	6.7
Retired	1017	39.0	519	44.1	293	34.6	108	37.4	35	30.2	62	34.6
Not employed/homemaker/student/on disability	413	15.8	112	9.3	128	15.1	58	20.1	56	48.3	59	33.0
**Education**, n (%)												
Some high school or less	288	10.7	107	9.0	77	8.8	50	16.6	19	15.3	35	18.5
High school/GED certificate	752	28.0	317	26.6	246	28.0	87	28.8	41	33.1	61	32.3
Some college/tech. school	840	31.3	358	30.0	272	31.0	111	36.8	43	34.7	56	29.6
College and above	806	30.0	411	34.5	283	32.2	54	17.9	21	16.9	37	19.6
**Income**, n (%)												
≤$20,000	620	28.1	168	18.0	177	24.2	119	45.3	77	70.6	79	47.0
$20,001-$50,000	809	36.7	330	35.3	283	38.6	104	39.5	25	22.9	67	39.9
≥$50,001	778	35.3	436	46.7	273	37.2	40	15.2	7	6.4	22	13.1
**Rurality**, n (%)												
In town	694	26.0	289	24.4	223	25.5	79	26.6	42	33.9	61	32.1
Rural area, neighbors close by	1359	50.9	619	52.2	454	51.8	149	50.2	49	39.5	88	46.3
Rural area, few neighbors close by	619	23.2	277	23.4	199	22.7	69	23.2	33	26.6	41	21.6
**Household size**, n (%)												
Lives alone	632	24.0	261	22.23	191	22.2	81	27.6	43	35.3	56	29.5
Lives with 1 other	1176	44.6	569	48.6	376	43.7	130	44.2	43	35.3	58	30.5
Lives with ≥2 others	341	29.1	341	29.1	293	34.1	83	28.2	36	29.5	76	40.0

**Table 6:** Survey Respondent Health Characteristic Descriptives by Well-being Profile (n = 2,720).

Profile	Total		Very high		High		Medium		Low		Mixed	
Variable	N/mean	%/SD	N/mean	%/SD	N/mean	%/SD	N/mean	%/SD	N/mean	%/SD	N/mean	%/SD
Sample size and descriptives, n (%)	2720	100.0	1210	44.5	886	32.6	305	11.2	127	4.7	192	7.1
PWI Score, Mean (SD)	7.6	1.9	9.1	0.6	7.4	0.6	5.4	0.7	2.6	1.0	5.3	0.9
Variable	N	%	N	%	N	%	N	%	N	%	N	%
**Food security**, n (%)												
Secure	1274	55.1	756	73.5	395	53.2	64	24.9	14	12.8	45	25.9
Insecure	1037	44.9	273	26.5	347	46.8	193	75.1	95	87.2	129	74.1
**Body mass index**, n (%)												
Healthy (18.5–24.9)	649	25.1	319	28.3	184	21.8	70	25.0	23	20.0	37	21.0
Overweight (25.0–29.9)	883	34.1	416	36.9	288	34.1	90	32.1	27	23.5	49	27.8
Obese (≥30.0)	1059	40.9	391	34.7	373	44.1	120	42.9	65	56.5	90	51.1
**Health status**, n (%)												
Poor/fair	686	25.4	126	10.5	222	25.3	121	39.8	93	73.8	124	65.3
Good/very good/excellent	2016	74.6	1077	89.5	657	74.7	183	60.2	33	26.2	66	34.7

#### Age, gender, and race.

The Low, Medium, and High well-being groups were significantly less likely to be age ≥65 than the Very High well-being group, with ORs=0.63 (95%CI: 0.48–0.82), 0.63 (95%CI: 0.48–0.82), and 0.58 (95%CI: 0.48–0.70), respectively. In addition, the Low well-being group was significantly less likely to be age ≥65 than the Medium well-being group, OR=0.62 (95%CI: 0.39–0.99). On gender, the Medium and Low well-being groups were significantly less likely to be male than the Very High profile, OR=0.67 (95%CI: 0.51–0.89) and OR=0.57 (95%CI: 0.37–0.88), respectively. There were similar differences for gender comparing High well-being to the Medium and Low well-being profiles (S2 Table). The Mixed well-being group was not significantly different from any other group on gender. For race, the Low, Medium, and High well-being groups were significantly less likely than the Very High well-being profile to be White, with ORs=0.43 (95%CI: 0.28–0.65), 0.42 (95%CI: 0.31–0.56), and 0.63 (95%CI: 0.50–0.78), respectively. The Mixed group was significantly more likely to be White than the Low and Medium well-being groups, OR=2.22 (95%CI: 1.29–3.82) and OR=2.27 (95%CI: 1.43–3.60), respectively.

#### Marital status.

The Very High well-being profile was significantly more likely to be married than the High, Medium, and Low well-being profiles, with ORs=1.32 (95%CI: 1.11–1.58), 2.81 (95%CI: 2.17–3.65), and 4.5 (95%CI: 2.99–6.76), respectively. The Very High well-being group reported the highest prevalence of married respondents (63.6%), and the Low well-being group had the lowest (28.0%). The Very High and High well-being groups were significantly more likely to be married than those in the Mixed well-being profile, OR=3.00 (95%CI: 2.18–4.12) and OR=2.27 (95%CI: 1.64–3.14), respectively.

#### Employment status.

There was a strong relationship between employment and well-being profile, with the greatest proportion of those not employed/homemakers/students/on disability in the Low well-being group (48.3%) and the smallest proportion in the Very High well-being group (9.3%). The High, Medium, and Low well-being profiles were significantly more likely to be not employed than the Very High well-being group, with ORs of 1.44 (95%CI: 1.08–1.93), 2.34 (95%CI: 1.59–3.44), and 13.03 (95%CI: 7.29–23.29), respectively. Additionally, the proportion of Mixed well-being profile members that were not employed (33.0%) was significantly greater than the Medium, High, and Very High profiles, with ORs=2.17 (95%CI: 1.31–3.59), 3.52 (95%CI: 2.28–5.44), and 5.07 (95%CI: 3.28–7.86), respectively (S2 Table).

#### Educational attainment.

Regarding education, the Very High and High well-being profiles showed statistically significant differences from the other well-being profiles. Compared to the Very High well-being profile, the Medium, Low, and Mixed well-being profiles were more likely to have some high school education or less, with ORs 3.56 (95%CI: 2.29–55.2), 3.48 (95%CI: 1.80–6.70), and 3.63 (95%CI: 2.19–6.04), respectively. The High well-being profile showed a similar pattern. There were similar patterns of significant differences between profiles for having a high school diploma or GED certificate, and for some college or technical school (S2 Table).

#### Income.

There was a strong positive relationship between income-level and well-being profile. Those in the highest income group reported the second-highest PWI score (mean=8.2) among all demographic subgroups. The High, Medium, Low, and Mixed profiles were significantly more likely than the Very High well-being profile to report an annual household income of ≤$20,000, with ORs of 1.68 (95%CI: 1.30–2.18), 7.72 (95%CI: 5.18–11.52), 28.54 (95%CI: 12.90–63.11), and 9.32 (95%CI: 5.63–15.44), respectively. There was a significant difference between nearly all well-being profiles at this income level, with 18.0% of the Very High well-being group at this income level, compared to 70.6% of Low well-being profile members. There was a similar pattern for incomes of $20,001-$50,000, although there were no significant differences between the Medium, Low, and Mixed well-being profiles.

#### Neighborhood type.

Low well-being profile members were significantly less likely to live in a rural area with neighbors close by than the Very High and High well-being profiles, OR=0.55 (95%CI: 0.35–0.84) and OR=0.57 (95%CI: 0.37–0.89) respectively. The Mixed well-being group was significantly less likely to live in a rural area with neighbors close by than those in the Very High well-being profile, OR 0.67 (95%CI: 0.47–0.96). There were no significant differences by well-being profile for those living in a rural area with few neighbors close by.

#### Household size.

The Low well-being group was significantly less likely to live in a two-person household than the Very High or High well-being groups, OR=0.46 (95%CI: 0.29–0.72) and OR=0.51 (95%CI: 0.32–0.80), respectively. The Mixed well-being group was also significantly less likely to live in a two-person household than those in the Very High and High well-being groups, OR=0.48 (95%CI: 0.32–0.71) and OR=0.53 (95%CI: 0.35–0.79), respectively.

#### Food insecurity, BMI, health status.

We observed consistent, strong relationships between health-related items and well-being profiles (Table 6). For food insecurity, there was a strong negative relationship with well-being profile, with significant differences between each of the four main well-being profiles and every other well-being profile (S2 Table). Prevalence of food insecurity ranged from (26.5%) in the Very High well-being profile to (87.2%) in the Low well-being profile. The Mixed well-being group reported similar levels of food insecurity (74.1%) as the Medium well-being profile (75.1%), and was significantly less likely to be food secure than the High and Very High profiles, OR=0.31, (95%CI: 0.21–0.44) and OR=0.13 [95%CI: 0.09–0.18), but significantly more likely to be food secure than the Low well-being profile, OR=2.37 (95%CI: 1.23–4.56). Food secure respondents reported the highest PWI score (mean=8.3) among all demographic subgroups. For BMI, the High, Medium, and Low well-being groups were significantly more likely than the Very High well-being group to be obese, with ORs 1.66 (95%CI: 1.32–2.09), 1.44 (95%CI: 1.04–2.01), and 2.10 (95%CI: 1.30–3.39), respectively). For health status, there were significant differences for nearly every well-being profile, with the greatest disparity of groupwise comparisons indicating the Low well-being profile was much more likely to report poor/fair health than the Very High well-being profile, OR=24.09 (95%CI: 15.55–37.33). Among those in the Low well-being group, 73.8% reported poor or fair health, much greater than the Medium (39.8%), High (25.3%), and Very High (10.5%) profiles (Table 6). About two-thirds of the Mixed well-being profile reported poor/fair health (65.3%).

## Discussion

With this study, we sought to identify sociodemographic and health factors associated with personal well-being in a sample of rural adults living in the state of Georgia. While few studies have considered rurality in assessments of well-being, this paper is, to our knowledge, the first to explore well-being in an exclusively rural population. We observed consistent differences in PWI scores and for each PWI domain across all sociodemographic categories, with characteristics that have historically conferred advantages in the U.S. generally being associated with greater well-being. This includes adults who were older, male, White, married, college-educated, and had relatively high-incomes. These findings are consistent with previous studies of well-being and related constructs in U.S. populations.

The Latent Profile Analysis revealed five distinct well-being profiles, with most respondents falling into the Very High or High well-being groups. The overall PWI score for our sample was 7.6, which is roughly equivalent to the High well-being profile, and comparable to normal distribution in western populations [31]. In the 2005 Behavioral Risk Factor Surveillance System survey, 5.6% of Americans reported low levels of life satisfaction [8], similar to the 4.7% of our sample comprising the low well-being profile. Given demographic and infrastructure differences between urban and rural communities, one might expect to find lower levels of well-being in rural communities. However, that expectation may underestimate the strengths, resources, and resilience of rural communities that may offset challenges. Beyond exploring well-being in a rural sample and whether it differs from urban communities, we were interested in exploring the data by degree of rurality. We found that those living in a rural area with neighbors close by (about half of our sample) reported slightly higher levels of well-being compared to those who lived in-town or in more rural areas. Additional research in other rural U.S. communities may help clarify the relationship between rurality and well-being. In addition, understanding if there are rural-urban/suburban differences in drivers of well-being between groups will be key.

Spirituality or religion was the highest rated PWI domain overall, among all four main well-being profiles, and among all demographic subgroups except young adults and those working full-time, for both of whom it ranked second highest. International data have shown that people who are active in religion are happier and report better health than those not actively religious [36]. U.S. data suggest that attending religious services is associated with living longer [37]. Although happiness and lifespan are not directly related to concepts of interest in this paper, this generally supports the notion that participation in religion is positively associated with self-reported health and well-being. While satisfaction with one’s spirituality or religion does not necessarily equate to religiosity, the consistently high level of satisfaction in this domain is noteworthy and may shed light on key factors for well-being in rural communities.

Some demographic traits showed complex relationships with well-being, such that different sociodemographic groups ranked higher or lower for some PWI domains than others. Age is a key example. Although adults age ≥65 were most likely to be in the High or Very High well-being profile, there was variation by age on key life domains. Older adults reported the highest levels of satisfaction for the PWI overall and all but two domains (including health where they tied with young adults aged 18–34), whereas young adults rated highest on personal relationships and feelings of safety. Notably, the middle-age group ranked lowest on the PWI overall and five domains, not reporting the highest rating on any domain. This finding is generally consistent with the U-shaped relationship between age and well-being observed in other studies [2,7–9]. This is a pattern that may be unique to rich English-speaking countries, versus other parts of the world that have shown declining well-being with age or no relationship [2]. Keyes [13,14] also found the highest levels of flourishing among older adults.

In general, there were strong positive relationships between well-being and income level, educational attainment, and employment status (being retired or working either full- or part-time compared to not working). Findings were consistent for the PWI score, individual well-being domains, and well-being profile. These findings are consistent with other studies that have linked greater levels of well-being with higher incomes [9,11], more years of education [8–10,13,14], and employment [8–10,14]. These relationships are likely the result of greater feelings of control in life, ability to make progress toward goals, and sense of meaning that money, an education, and a job may confer [38].

As discussed above, socioeconomically advantaged groups tended to comprise the Very High and High well-being profile groups, whereas those from more disadvantaged groups occupied the Medium, Low, and Mixed well-being profiles. As suggested in the name, the Mixed well-being profile group demonstrated a different pattern than the other well-being profiles. They were most similar to the Medium well-profile, but certain well-being domains appeared closer to the Low or High well-being profile groups. On race and gender, however, the Mixed well-being profile was closest to the Very High well-being profile. Both the Very High and Mixed profiles had a higher proportion of group members who were White and male compared to the other profiles (although the Mixed group was not significantly different from other profiles on gender). As documented in prior research [39], it is possible that the intersectional identity advantages conferred by being both White and male are responsible for higher ratings of feeling safe compared to those in the Low and Medium well-being groups who were more likely to be Black and female. Future research should further explore the relationship between specific sociodemographic factors and ratings of well-being, and underlying causes to the extent possible.

In our study, well-being was strongly associated with specific health factors. We found that the proportion of those in the Low well-being profile that reported poor or fair health was much greater than the Medium, High, and Very High well-being profiles. Behavioral Risk Factor Surveillance System data from 2005 and 2009 had similar findings, with strong correlations between poor/fair health and life dissatisfaction among adults [8] and women ages 18–44 [9]. We also found that the Very High well-being profile group was significantly less likely to be obese than all other well-being profiles. This is consistent with past research that has found obesity to be about 1.5 times more likely among those reporting life dissatisfaction [8]. Another study found that BMI measured as a continuous variable was about two points lower among those who were flourishing than those who were not [14]. We are not aware of other research on well-being and food insecurity but given the strong associations between well-being and economic factors, the inverse relationship we observed seems plausible yet may need further exploration.

### Limitations

Several limitations of this work should be considered. First, as with most studies on well-being, we used self-reported, cross-sectional survey data to explore relationships with sociodemographics, but cannot assess causality. Second, although surveys were mailed to random households in each county with robust follow-up, our sample of respondents are proportionally older, more likely to be female, White, retired, and low-income to compared to U.S. Census data from participating counties. Furthermore, the LPA revealed a high prevalence of respondents falling into the Very High well-being profile, with possible explanations including the exclusively rural sample, high proportion of those living in a rural area with close neighbors, and satisfaction with religion/spirituality, which may limit generalizability and/or suggest bias in our sample. In addition, our response rate is somewhat low (26.2%), yet consistent with recommendations for surveillance efforts in rural communities [40]. Finally, these data were collected before the COVID-19 pandemic, and while that serves as a useful baseline for the broader initiative evaluation, data suggest sharp national declines in both current and anticipated life satisfaction in 2020 [41] and that rural communities may have been especially vulnerable to the effects of the pandemic [42].

## Conclusion

To address well-being, we need to reach a common understanding of what it is and how it differs from health. Perhaps Healthy People 2030’s elevation of well-being to a desired outcome for Americans will spur the development of a clear definition and operationalization of well-being domains, both objective and subjective. Furthermore, research is needed to identify the best measure(s) to assess well-being, factors that help achieve it, and those that serve as barriers. A critical gap in the literature pertains to directionality of the relationship between sociodemographics and well-being. As Meit & Knudson [18] suggested, rural communities provide cost-effective opportunities to test innovative intervention strategies. Multisectoral coalitions, such as those involved in this initiative, collaborating with researchers and evaluators may be an effective mechanism for identifying factors that influence well-being and health. Interventions on SDOH may help determine successful approaches to promote well-being and health and reduce socioeconomic inequities. Evidence produced by such research may ultimately inform local, state, and national policy goals to improve Americans’ well-being and health. Research and policy should consider impacts on underserved populations to ensure equity in implementation, with the aim of ensuring well-being is achieved equitably across sociodemographic groups.

## Supporting information

S1 Table Latent profile analysis model fit indicators. (DOCX)

S2 Table Latent profile analysis groupwise odds ratios. (XLSX)
