# Supplementary material for: Well-being inequities in eleven rural Georgia communities: A latent profile analysis
Source: PLoS One. 2025 Apr 29;20(4):e0320222. doi: 10.1371/journal.pone.0320222 (PMC12040219; doi:10.1371/journal.pone.0320222)
Supplement: S1 Table — (DOCX) [file pone.0320222.s001.docx]

S1 Table. Latent Profile Analysis Model Fit Indicators

|  | **Number of profiles** | | | | | | |
| --- | --- | --- | --- | --- | --- | --- | --- |
| **Model Fit measure** | **2** | **3** | **4** | **5** | **6** | **7** | **8** |
| **Akaike Information Criterion** | 89316.40 | 86813.86 | 85738.18 | 85224.80 | 84902.15 | 84452.26 | 84089.40 |
| **Bayesian Information Criterion** | 89464.11 | 87014.74 | 85992.24 | 85532.03 | 85262.56 | 84865.85 | 845561.7 |
| **Adjusted Bayesian Information Criterion** | 89384.68 | 86906.71 | 85855.61 | 85366.81 | 85068.75 | 84643.43 | 84305.16 |
| **Entropy** | 92.6 | 91.2 | 86.0 | 86.7 | 86.1 | 85.1 | 85.8 |
| **Lo-Mendell-Rubin adjusted Likelihood Ratio Test** | <.0001 | 0.0004 | 0.001 | 0.003 | 0.75 | 0.24 | 0.26 |
